# The long-term dynamics of *Campylobacter* colonizing a free-range broiler breeder flock: an observational study

**DOI:** 10.1111/1462-2920.12415

**Published:** 2014-03-11

**Authors:** Frances M Colles, Noel D McCarthy, Carly M Bliss, Ruth Layton, Martin C J Maiden

**Affiliations:** 1 The Department of Zoology, University of Oxford, South Parks RoadSouth Parks Road, Oxford, OX1 3PS, UK; 2 The Food Animal Initiative, The John Krebs Field StationWytham, Oxford, OX2 8QJ, UK

## Abstract

A free-range broiler breeder flock was studied in order to determine the natural patterns of *Campylobacter* colonization over a period of 63 weeks. *Campylobacter* sequence types (STs) were not mutually exclusive and on average colonized only 17.7% of the birds tested at any time. *Campylobacter* STs typically reached a peak in prevalence upon initial detection in the flock before tailing off, although the ST and antigenic *flaA* short variable region in combination were stable over a number of months. There was evidence that, with a couple of exceptions, the ecology of *C. jejuni* and *C. coli* differed, with the latter forming a more stable population. Despite being free range, no newly colonizing STs were detected over a 6-week period in autumn and a 10-week period in winter, towards the end of the study. There was limited evidence that those STs identified among broiler chicken flocks on the same farm site were likely to colonize the breeder flock earlier (*R^2^* 0.16, *P* 0.01). These results suggest that there is natural control of *Campylobacter* dynamics within a flock which could potentially be exploited in designing new intervention strategies, and that the two different species should perhaps be considered separately.

## Introduction

Contaminated chicken meat has been identified as a major source of human campylobacteriosis using case-control and genetic attribution studies (Wilson *et al*., 1998; Sheppard *et al*., 2009; Tam *et al*., 2009; Doorduyn *et al*., 2010; Fajo-Pascual *et al*., 2010). Reducing the prevalence of *Campylobacter* among broiler flocks therefore remains a high priority among policy makers. On-farm biosecurity measures are the main defence in excluding *Campylobacter* from housed flocks, but they commonly break down, and an average of 71.2% of broiler flocks in the European Union are positive at slaughter (Newell and Fearnley, 2003; EFSA, 2010). A more detailed understanding of the ecology of *Campylobacter* among commercial flocks is needed to aid the design of more effective intervention methods; however, broiler chickens are slaughtered at an immature age giving little scope to study interactions of the organism and host over time.

Once detected in a flock, *Campylobacter* can be isolated in very high numbers from most of the other birds within a few days, and also from their immediate environment (Lindblom *et al*., 1986; Shanker *et al*., 1990; Shreeve *et al*., 2000). Colonized flocks usually remain positive until slaughter, where high levels of the organism in intestinal contents exacerbate contamination of the meat product during automated processing (Jacobs-Reitsma *et al*., 1994; Johnsen *et al*., 2006). Studies of older free-range, organic and breeder birds in particular, as well as turkeys, have noted the presence of multiple *Campylobacter* strains, and a change in the dominant strain at around 30 days of age (Wallace *et al*., 1998; Schouls *et al*., 2003; Hook *et al*., 2005; Bull *et al*., 2006; De Cesare *et al*., 2008; Kudirkiene *et al*., 2010). It is thought that *Campylobacter* strains differ in their ability to colonize the intestinal tract, and ‘hyper-colonizing’ strains have been identified that are consistently able to replace others in experimental birds (Ringoir and Korolik, 2003; Calderon-Gomez *et al*., 2009). Another study points towards a more general process, whereby strains are randomly lost or transmitted between co-housed birds (Grant *et al*., 2005), and others suggest host immunity, which may be strain specific, may limit colonization by *Campylobacter* over time (Lindblom *et al*., 1986; Skanseng *et al*., 2007). Raised antibody levels and pro-inflammatory responses can be detected among chickens in response to *Campylobacter*, but the lack of overt disease means that it is generally regarded as a commensal of the chicken intestine (Cawthraw *et al*., 1994; Hendrixson and DiRita, 2004; Janssen *et al*., 2008; Smith *et al*., 2008).

The aim of this study was to observe the population dynamics of *Campylobacter* colonizing a broiler breeder flock for an extended period of time in order to gain a better understanding of chicken/*Campylobacter* interactions. Due to the nature of the study in a commercial setting, it was not possible to use standard sampling methods applicable in controlled experimental settings. It was necessary to use swabs of the cloacal opening rather than post-mortem caecal contents samples, to enable continued sampling of the same individual birds. The swabs gave an 88.9% sensitivity level, and equivalent detection of *Campylobacter* diversity, in comparison to caecal contents samples during method validation (Colles *et al*., 2011). All *Campylobacter* isolates were genotyped by multi-locus sequence typing (MLST) of fragments of seven house-keeping genes, giving definitive identification of the *Campylobacter* species and strain (Dingle *et al*., 2001a).

## Results

### Genetic diversity

**Fig 1:**
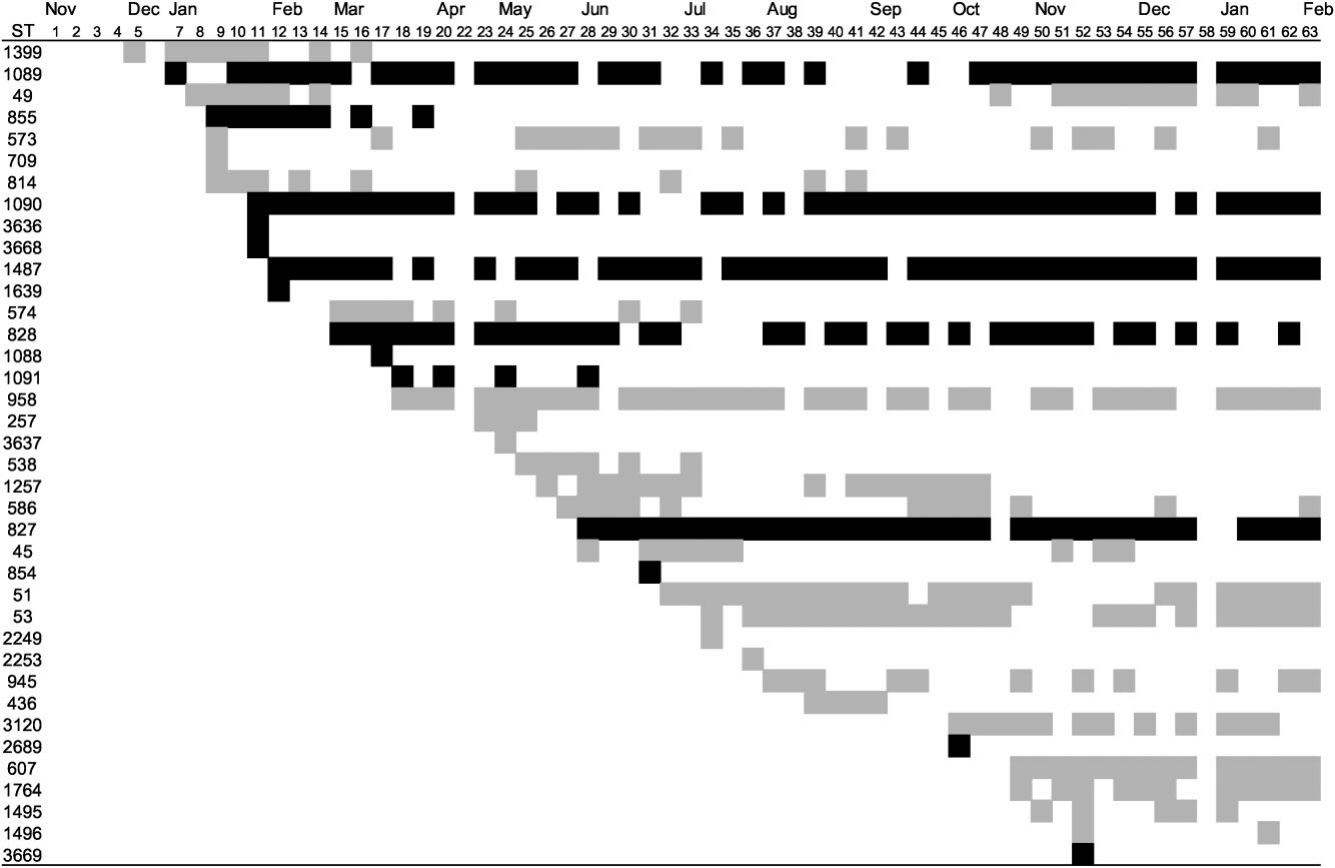
The distribution of *Campylobacter* STs isolated from the free-range broiler breeder flock over the study period, shown in the order in which they colonized the flock. No samples were collected in weeks 21, 22 and 58.

**Fig 2:**
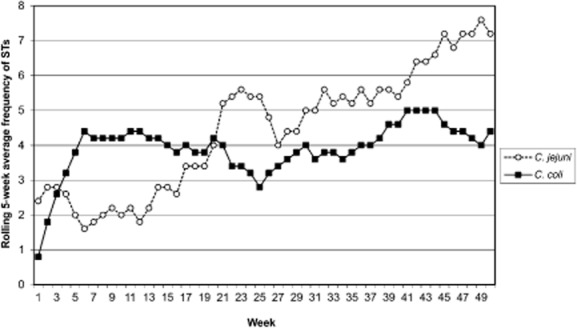
Graph showing the 5-week rolling average frequency of *C. jejuni* and *C. coli* isolated from the free-range broiler breeder.

A total of 1738 *Campylobacter* isolates were obtained from 4105 samples giving an average prevalence rate of 42.3% over the course of the study. Of these, 887 (51.0%) were *C. jejuni* and 851 (49.0%) were *C. coli* (Table 1 and Supporting Information Table S1). The *C. jejuni* isolates comprised 25 sequence types (STs) of which 17 were assigned to 12 clonal complexes, and eight STs remained unassigned. The *C. coli* isolates comprised 14 STs with 11 assigned to two clonal complexes and three unassigned. Eight of the 39 (20.5%) STs accounted for 66.8% of the isolates, with the remainder accounting for less than 5% of isolates each. The STs that were most commonly isolated were the *C. coli* ST-1487 (14.3% of isolates) and the *C. jejuni* ST-958 (11.7% of isolates). Ninety-two ST-*flaA* short variable region (SVR) combinations were identified in total, with individual STs being associated with between one and eight (and a mean of two) *flaA* SVR types over the course of the study (Supporting Information Table S1). Between one and nine clonal complexes, and one (week 5) and 14 STs (week 52) were isolated from the flock in any given week (Fig. 1). A 5-week rolling average frequency analysis (Fig. 2) demonstrated that the number of *C. jejuni* STs isolated from the flock showed a general increase over time, while the number of *C. coli* STs isolated from the flock each week rose quickly and was then relatively stable at a level of between three and five STs. Interrogation of the PubMLST database (http://pubmlst.org/campylobacter/, campylobacter (accessed on 03.01.2013) recording over 6000 allelic profiles and more than 22 000 isolates, revealed that many of the STs isolated from the free-range broiler breeder flock have previously been isolated from chicken sources, are distributed on a worldwide basis and have been isolated over at least a decade (Table 1). Six of the 39 (15.4%) STs were unique to the study, all of which were isolated four times or fewer.

**Table 1:** Colonization parameters of the *Campylobacter* genotypes, shown in the order in which the free-range broiler breeder flock was colonized

					Previous isolations^a^		
					
ST	No. sampling occasions present	Isolation period (days)	Time to infection peak (days)	Birds colonized at infection peak (%)	Host source^b^	Year of first record	Distribution^b^
1399	8	86	35	20	C,H	2001	UK
1089^c^	40	404	35	16	C	2003	UK
49	17	395	21	13	C,H,F,E	1983	WW
855^c^	9	79	7	52	C,H	2001	Europe
573	19	263	63	3	C,H	2000	UK
709	1	1	na	0.5	E	2002	UK
814	9	224	21	9	C,H	2001	UK
1090^c^	42	374	1	21	C	2003	UK
3636^c^	1	1	na	0.5	U	2004	UK
3668^c^	1	1	na	0.5	U	2004	UK
1487^c^	44	365	1	27	C,H	1998	WW
1639^c^	1	1	na	0.5	H	1999	WW
574	8	127	7	16	C,H,E	1999	WW
828^c^	32	336	42	6	C,H,F	2002	WW
1088^c^	1	1	na	0.5	C,H	2003	UK
1091^c^	4	71	1	1	U	2004	UK
958	38	355	28	20	C	2003	UK
257	3	14	7	11	C,H.F,E	1990	WW
3637	1	1	na	0.5	U	2004	UK
538	6	56	7	7	H,E,O	2001	WW
1257	15	147	49	6	C,E	2002	Europe
586	12	260	21	9	C,H,F	2000	Europe
827^c^	33	253	28	13	C,H,F,E	2000	WW
45	9	182	49	10	C,H,F,E	1982	WW
854^c^	1	1	na	0.5	C,H,F,E	2002	WW
51	24	210	28	21	C,H	1982	WW
53	23	210	25	13	C,H,F,E	1984	WW
2249	1	1	na	0.5	C,H	2001	UK
2253	1	1	na	0.5	–	2004	UK
945	11	190	7	7	C,H,E	2002	Europe
436	4	21	1	4	H,E	1998	WW
3120	12	105	7	9	U	2004	UK
2689^c^	1	1	na	0.5	C	2004	UK
607	14	106	7	25	C,H	2000	WW
1764	11	106	21	6	E	2003	Europe
1495	5	64	1	1	C	2004	UK
1496	2	63	na	0.5	C	2004	UK
3669^c^	1	1	na	0.5	U	2004	UK
1223^d^	1	1	na	na	E	1999	WW

a Source of data *Campylobacter* PubMLST database http://pubmlst.org/campylobacter/.

b C = chicken, H = human disease, F = farm animals (cattle, sheep, pigs), E = environmental (water, wild birds), U = unique to the study at present, O = other (horse), – = unknown, na = not applicable, WW = world wide.

c *C. coli* genotypes.

d This ST was isolated from a small group of new male birds before they were added to the main flock.

### Prevalence, persistence and infectivity of genotypes

**Fig 3:**
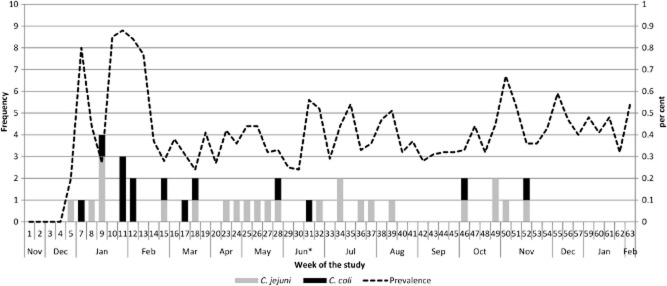
*Campylobacter* prevalence versus the number of STs newly identified among the broiler breeder flock.Dark shading = *C. coli* isolates, grey shading = *C. jejuni* isolates. *Just one ST, ST-51 was newly isolated from both flock types in the same week (week 32) in June.

There was a succession of *Campylobacter* STs isolated from the broiler breeder flock, but they were not mutually exclusive, and they varied in duration of detection (Fig. 1). The length of time between which an ST was newly isolated from the flock increased towards the end of the study, with no STs newly detected during September 2004, December 2004, January 2005 or the first week in February 2005 (Fig. 3). There was no evidence that the overall prevalence of *Campylobacter* in the flock was associated with STs being newly isolated from the flock, when tested at the same time, 1, 2 or 3 weeks post identification, or if the initial bloom in prevalence prior to week 15 was removed from the analyses (*R^2^* < 0.001 to 0.092, *P* 0.024–0.999).

For the STs detected on more than 1 day, the period of isolation ranged from 14 to 404 days, with a mean of 115.4 days for *C. jejuni*, 268.9 days for *C. coli* and 130.2 days for a ST-*flaA* SVR type in combination (Table 1). The time taken by an ST to reach the maximum number of shedding birds ranged from 1 to 63 days, with a mean of 20. The number of chickens colonized at the peak of infection by a particular ST varied from 1 (0.5%) to 52 (26.0%) of the birds tested, with an average of 13.3 (17.7%). The frequency of all but two STs isolated on more than five occasions expanded on entering the flock, with the exceptions being STs 1090 and 1487. The maximum proportion of the flock colonized by a particular ST was on average four times (range 1 to 22.75 times) greater in the first half of the period of isolation compared with the second half. STs 1090 and 1487 did not increase in prevalence following primary isolation, but the initial point of entry to the flock may have been undetected and the early expansion not therefore shown. ST-45 and ST-49 were the only two examples of an ST that may have potentially re-colonized the flock after an extended period of being undetectable (15 and 33 weeks respectively), with both being isolated at a frequency lower than the peak seen on initial colonization.

### Comparison with contemporary broiler flocks on the same farm

*Campylobacter* STs from the broiler breeder flock were compared with those isolated from a rolling production of broiler flocks aged 56 days on the same farm for almost a year previous to the breeder flock, and also during a 36-week period of overlap (Colles *et al*., 2008b; 2011[Bibr b7],[Bibr b9]). Of the 39 STs isolated from the breeder flock, and 59 STs (from 2041 isolates) isolated from the broiler flocks, 16 STs (eight *C. jejuni* and eight *C. coli*) overlapped. Seven of the 16 (43.8%) STs isolated from both flock types were present simultaneously, and there was some correlation in the order of succession of both breeder flock and farm site (*R^2^* 0.301, *P* 0.023). There was some evidence that those STs previously isolated on the farm were more likely to colonize the breeder flock earlier (*R^2^* 0.158, *P* 0.013). Only ST-51 was newly isolated from both flock types in the same week.

In general, STs that were isolated over long time periods on the farm showed no correlation with those that were able to persist for a long time within the broiler breeder flock (*R^2^* 0.034, *P* 0.448). In contrast to the broiler breeder flock, there was no evidence that *C. jejuni* and *C. coli* differed in mean isolation period on the farm site (126 compared with 145 days when STs isolated only once were removed from the analysis).

## Discussion

We present results from an observational study of extended colonization dynamics of *Campylobacter* among a free-range broiler breeder flock that, by definition, was continually exposed to environmental sources of contamination. Despite this, all but six *Campylobacter* STs isolated from the broiler breeder flock during the study had previously been isolated and deposited on the PubMLST database (http://pubmlst.org/campylobacter/); the majority associated with chicken and human disease sources. The ratio of STs to the number of isolates was 0.02 (39:1738); an order of magnitude lower than that seen among wild geese, 0.23 (38:166) and starlings, 0.27 (75:277) sampled on the same farm, implying that the *Campylobacter* genotypes colonizing the flock over a year were significantly less diverse than those seen in much smaller isolate collections from wild bird species. (Colles *et al*., 2008a; 2009[Bibr b6],[Bibr b8]). The most likely explanation is that a domestically reared chicken flock comes into contact with fewer sources of *Campylobacter* compared with the wild birds, but other factors such as the genetic diversity and mixing of the host population, age, immune status and stocking density are likely to be of importance. Levels of diversity similar to that seen in wild birds can be obtained from retail chicken meat, where a much greater number of chicken flocks from a variety of farms and producers are sampled (Sheppard *et al*., 2010).

The general pattern of infection for all STs isolated over several weeks was consistent with a peak in the number of chickens colonized shortly after an ST was first detected, followed by reduced frequency in subsequent weeks. Nearly all of the STs isolated in any number had the same pattern, irrespective of what stage they entered the flock, which is consistent with strain-specific host immunity being important in controlling *Campylobacter* population dynamics. Although there is debate as to whether *Campylobacter* is commensal within the chicken intestinal tract, raised levels of *Campylobacter* specific antibodies and a pro-inflammatory response has been demonstrated (Cawthraw *et al*., 1994; Smith *et al*., 2008). Observations from this study are consistent with the results of an experimental study demonstrating that strain-specific immune response by the host, rather than background gut microbiota, was important in controlling a shift in strains during the infection period (Skanseng *et al*., 2007).

There was evidence that *C. coli* STs formed a more stable population within the flock, persisting over many weeks at a consistent rate of prevalence, in contrast to *C. jejuni* STs, which in general were isolated over shorter periods of time and colonized a greater proportion of the flock at the peak of infection. Notable exceptions to this general rule were the *C. jejuni* ST-958 isolated over a 45-week period and *C. coli* ST-855 isolated over an 11-week period. *Campylobacter. coli* is sometimes seen to succeed *C. jejuni* among broiler flocks, particularly those that are free range or slightly older, with 35 days of age being a critical time point for a change in dominant *Campylobacter* strain, perhaps as a result of competitive advantage or due to host-related changes (El-Shibiny *et al*., 2005; 2007[Bibr b17],[Bibr b18]). Results from this study demonstrate that changes continue to occur over many months and notwithstanding the prolonged presence of *C. coli*, *C. jejuni* STs were still able to colonize the broiler breeder flock. The different species characteristics provide an opportunity to investigate cellular mechanisms of persistence upon which future intervention strategies such as vaccination may potentially be developed. Additionally, more information is needed regarding the role of *C. coli* in broiler chickens; it is possible that removing a stable *C. coli* population may be detrimental in allowing more virulent *Campylobacter* strains to access a particular niche instead. Small fluctuations in prevalence of an ST may result from limitations in sampling sensitivity, however, ST-45, undetected for 15 weeks, and ST-49, undetected for 33 weeks between periods of isolation, may be two examples whereby the flock has become re-colonized. For both STs, an initial rise in prevalence was absent during the second period of detection. The pattern of colonization by individual STs and the life span of the broiler flock being just over a year meant that little evidence for seasonal variation of *Campylobacter* genotypes, previously demonstrated for certain STs such as ST-45, could be established with this data set (Sopwith *et al*., 2008; Cody *et al*., 2012).

It might be expected that *Campylobacter* genotypes already existing on the farm site among free-range broiler flocks would be early colonizers of the breeder flock, either by direct transfer or via a shared external source. The results indicate that between 25.6% and 41.0% (10–16/39) of STs were common to both flock types, depending on whether or not STs that were present on the farm 6 to 12 months prior to the broiler breeder flock were included in the calculation. There was only one ST, ST-51, that was newly detected among both flock types simultaneously, suggesting that perhaps the flocks being tended by different farm staff was sufficient to prevent a greater carry-over of *Campylobacter* genotypes between flock types. Similarly, the fact that *C. jejuni* STs were detected only for short time periods among the broiler breeder flock but for long time periods on the farm site suggests that host factors are important in controlling *Campylobacter* population dynamics.

While it may be hypothesized that STs isolated only rarely from the flock may be novel variants, this did not appear to necessarily be the case, with seven of the 11 STs that were isolated only once in the study, having been identified previously on the PubMLST database, two with worldwide distribution, and one 4 years previous to this study. It was noteworthy that ST-1223, introduced to the main flock by the addition of new male birds, was not recovered from the other birds afterwards, a possible reason being that it is from a clonal complex (CC-1275) strongly associated with wild birds and perhaps less able to compete with other STs already present (Griekspoor *et al*., 2013).

The detection of STs that were new to the flock occurred sporadically, with a single ST being isolated on 60% (15/25) of occasions, and a maximum of four being isolated on the other occasions. In general, more STs were newly detected during winter and spring months at the start of the study, with no new STs detected in the months of September, January or February towards the end of the study. Overall *Campylobacter* prevalence in the breeder flock was not correlated with identification of new STs either within the flock. In common with previous findings (Colles *et al*., 2011), these results suggest that long-term population dynamics of *Campylobacter* colonization are independent of meteorological variables. Other factors which may influence fluctuations in ST prevalence include; the general dynamics involved with spreading from bird to bird; shedding status of the bird and associated changes in the gut microenvironment; inter-strain competition; strain-specific bacteriophage types which can only increase in number when the target reaches a critical level; and particularly with STs that may be seasonally variable, changes associated with the source of infection (Conlan *et al*., 2007; Cairns *et al*., 2009).

In conclusion, results from this study indicate that: (i) *C. jejuni* and *C. coli* may behave differently in terms of virulence and should perhaps be considered separately in terms of monitoring and in the design of on-farm interventions and (ii) that there was natural control of *Campylobacter* dynamics within a broiler breeder flock.

## Experimental procedures

### *Campylobacter* isolates

A free-range broiler breeder flock of 500 birds was sampled for *Campylobacter* on a weekly basis for 63 weeks between 2003 and 2005; further details of the flock have been previously published (Colles *et al*., 2011). Twenty-five fresh faecal samples were collected when the birds were aged 1 to 7 weeks, and 75 swabs of the cloacal opening were collected per week from the age of 8 weeks upwards. From 14 weeks of age, sampling was restricted to randomly selected birds within a cohort of 200 that were labelled individually with leg rings. All samples were cultured within 2 h of collection directly onto mCCDA (PO0119A Oxoid Ltd, Basingstoke, UK) and incubated in a microaerobic atmosphere at 42°C for 48 h. Single presumptive *Campylobacter* colonies were subcultured onto Columbia blood agar (PB0122A Oxoid Ltd, Basingstoke, UK) and incubated for a further 48 h at 42°C in a microaerobic atmosphere. Chromosomal DNA was extracted by boiling a cell suspension in PBS for 10 min and removing the sediment by centrifugation at 13 000 r.p.m. for 5 min or by using the method for rapid DNA extraction and the commercial IsoQuick nucleic acid extraction kit (ISC Bioexpress, Kaysville, UT).

Data from 1027 *Campylobacter* isolates that had been previously been isolated from 43 free-range broiler flocks reared on the same farm site as the broiler breeder flock at Wytham, Oxfordshire were used for comparison. The flocks were kept on a rolling production cycle, and between 10 and 100 cloacal swab samples were collected on a weekly basis from flocks that were 56 days of age. Further details of the flocks have been published previously (Jones *et al*., 2007; Colles *et al*., 2008b).

### Nucleotide sequence typing

Portions of seven housekeeping genes were sequenced using previously published protocols and primers (Meinersmann *et al*., 1997; Dingle *et al*., 2001b; 2002[Bibr b13],[Bibr b14]; Miller *et al*., 2005). The nucleotide extension reaction products were detected on an ABI Prism 3730 automated DNA analyser and assembled using methods described previously. Sequence types (STs) were assigned using the *Campylobacter* PubMLST database (http://pubmlst.org/campylobacter), which in addition uses an automated script to group related STs into clonal complexes on the basis of sharing four or more alleles with the previously identified central genotype.

### Statistical analyses

Regression analysis was used to test relationships between the order of succession of *Campylobacter* STs on the farm (among broiler flocks kept on rolling production) and in the broiler breeder flock; the period of isolation of *Campylobacter* STs on the farm site compared with the broiler breeder flock and the prevalence of *Campylobacter* within the flock compared with detection of STs new to the flock. The following were analysed as binary variables; presence or absence of a given ST on the farm site prior to detection in the broiler breeder flock and presence or absence of a ST newly identified in the broiler breeder flock. The isolation period for STs were considered separately where they were undetected or absent for 6 or more weeks, and the longest period for each ST included in the analyses. ‘Infection peak’ was defined as the maximum number of chickens shedding a given *Campylobacter* ST in a week. The 5-week rolling average graph for frequency of *C. jejuni* and *C. coli* graph was constructed by averaging the frequency of each species for each time point of 5 weeks in order to smooth out short-term fluctuations and clarify longer term trends. Regression analyses were performed using the STATA data analysis and statistical software package (StataCorp LP, College Station, TX, USA).

## Supporting information

**Table S1.** The *C. jejuni* and *C. coli* genotypes isolated from the free-range broiler flock throughout the study. ^a^C = central genotype, SLV = single locus variant, DLV = double locus variant, TLV = triple locus variant, na = not applicable, nt = not typed. ^b^ST-1223 was isolated from a small group of male birds before they were introduced to the main flock. ^c^*C. coli* genotypes.
