# Supplementary material for: The long-term dynamics of Campylobacter colonizing a free-range broiler breeder flock: an observational study
Source: Environ Microbiol. 2014 Mar 11;17(4):938–46. doi: 10.1111/1462-2920.12415 (PMC4390391; doi:10.1111/1462-2920.12415)
Supplement: Supplementary file 1 — Table S1. The C. jejuni and C. coli genotypes isolated from the free-range broiler flock throughout the study. aC = central genotype, SLV = single locus variant, DLV = double locus variant, TLV = triple locus variant, na = not applicable, nt = not typed. bST-1223 was isolated from a small group of male birds before they were introduced to the main flock. cC. coli genotypes. [file emi0017-0938-sd1.docx]

Supplementary data:

**Table 1.** The *C. jejuni* and *C. coli* genotypes isolated from the free-range broiler flock throughout the study. ^a^C=central genotype, SLV=single locus variant, DLV=double locus variant, TLV=triple locus variant, na=not applicable, nt=not typed. ^b^ST-1223 was isolated from a small group of male birds before they were introduced to the main flock. ^c^*C. coli* genotypes.
